# Anticipating Xenogenic Pollution at the Source: Impact of Sterilizations on DNA Release From Microbial Cultures

**DOI:** 10.3389/fbioe.2020.00171

**Published:** 2020-03-13

**Authors:** David Calderón-Franco, Qingnan Lin, Mark C. M. van Loosdrecht, Ben Abbas, David G. Weissbrodt

**Affiliations:** Department of Biotechnology, Delft University of Technology, Delft, Netherlands

**Keywords:** sterilizations, DNA release, xenogenic pollution, DIY biology, antimicrobial resistances

## Abstract

The dissemination of DNA and xenogenic elements across waterways is under scientific and public spotlight due to new gene-editing tools, such as do-it-yourself (DIY) CRISPR-Cas kits deployable at kitchen table. Over decades, prevention of spread of genetically modified organisms (GMOs), antimicrobial resistances (AMR), and pathogens from transgenic systems has focused on microbial inactivation. However, sterilization methods have not been assessed for DNA release and integrity. Here, we investigated the fate of intracellular DNA from cultures of model prokaryotic (*Escherichia coli*) and eukaryotic (*Saccharomyces cerevisiae*) cells that are traditionally used as microbial chassis for genetic modifications. DNA release was tracked during exposure of these cultures to conventional sterilization methods. Autoclaving, disinfection with glutaraldehyde, and microwaving are used to inactivate broths, healthcare equipment, and GMOs produced at kitchen table. DNA fragmentation and PCR-ability were measured on top of cell viability and morphology. Impact of these methods on DNA integrity was verified on a template of free λ DNA. Intense regular autoclaving (121°C, 20 min) resulted in the most severe DNA degradation and lowest household gene amplification capacity: 1.28 ± 0.11, 2.08 ± 0.03, and 4.96 ± 0.28 logs differences to the non-treated controls were measured from *E. coli*, *S. cerevisiae*, and λ DNA, respectively. Microwaving exerted strong DNA fragmentation after 100 s of exposure when free λ DNA was in solution (3.23 ± 0.06 logs difference) but a minor effect was observed when DNA was released from *E. coli* and *S. cerevisiae* (0.24 ± 0.14 and 1.32 ± 0.02 logs differences with the control, respectively). Glutaraldehyde prevented DNA leakage by preserving cell structures, while DNA integrity was not altered. The results show that current sterilization methods are effective on microorganism inactivation but do not safeguard an aqueous residue exempt of biologically reusable xenogenic material, being regular autoclaving the most severe DNA-affecting method. Reappraisal of sterilization methods is required along with risk assessment on the emission of DNA fragments in urban systems and nature.

## Graphical Abstract

Workflow overview of the study.

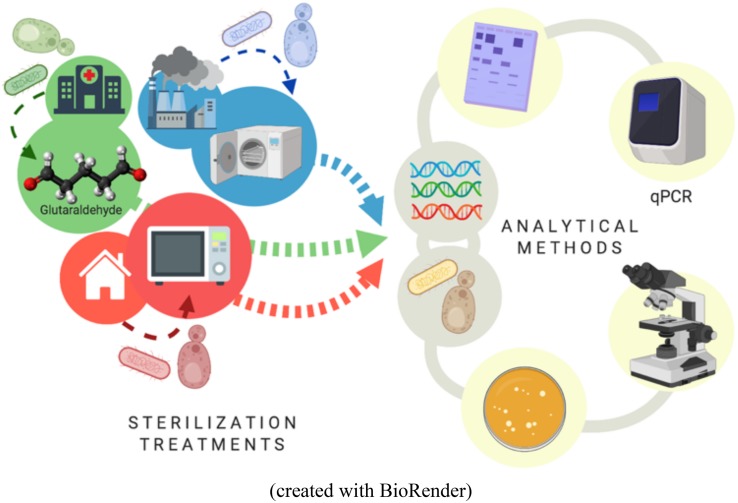

## Introduction

The rapid development of gene-editing tools together with the broad applications of genetically modified organisms (GMOs) have triggered biosafety concern on the hazard composed by the dissemination of unwanted DNA into the environment after sterilization (Simmon et al., 2004). Concerns have been risen on the emission of xenogenic and mobile genetic elements that may carry antibiotic resistance genes (ARGs) or pathogenicity, and their transport across urban waterways through wastewater treatment plants into nature (Bouki et al., 2013; Miller et al., 2016). Novel CRISPR-Cas technologies propel the engineering of microorganisms out of industry boundary with do-it-yourself (DIY) kits available at kitchen table. Uncontrolled, diffuse emission of GMO materials via domestic waste streams could be a threat.

Current sterilization methods are proven to efficiently inactivate microorganisms. However, a key knowledge gap remains on their impact on DNA and its potential release into industrial, clinical, and domestic sewage. Common methods used to treat industrial broths, healthcare equipment and surfaces, and domestic waste primarily involve autoclaving, glutaraldehyde, and microwaving, respectively.

Several studies have shown how DNA present in food products react with different sterilization procedures (Debode et al., 2007; Gryson, 2010; Bergerová et al., 2011). Treatments such as irradiation and autoclaving affect DNA in meat products or edible seeds by decreasing the total DNA content as well as causing DNA fragmentation, degradation and denaturation (Maity et al., 2009; López-Andreo et al., 2012). However, the impact highly depends on the cell type, the sterilization method, and the process conditions.

Temperature, pressure, pH, and sterilization times significantly exert effects on DNA quality. For instance, temperatures over 100°C have resulted in significant DNA strand clipping and irreversible loss of secondary structure (Gryson, 2010). Normal autoclaving (121°C between 5 and 20 min) of food and crops did not impeded it to be available for PCR amplification (Takeshi et al., 2003; Debode et al., 2007; Gryson, 2010).

Microwaving is commonly used in kitchen procedures such as water boiling and food heating (Kim et al., 2008; Fang et al., 2011). It has been suggested to effectively kill bacteria, yeast, and molds on kitchen sponges (Sharma et al., 2009). Microwaves at frequencies of 2450 MHz have been used to sterilize soil due to its ability to inhibit nitrification and sulfur oxidations (Ferriss, 1984; Trevors, 1996; Woo et al., 2000). It has also been used in laboratory settings for pharmaceutical glass vials, culture media, or clinical specimens sterilization (Akşen et al., 2004). The thermal effect mechanism is based on the absorption of microwave heat energy by the cell constituents, which leads to fast vibrations of cell membrane lipids resulting in the emergence of pores (Jankovic et al., 2014). These pores may cause leakage of vital intracellular molecules being able to cause cell death (Jankovic et al., 2014). High temperatures denature cellular biomolecules such as proteins, which may also be a reason of cells lysis (Karni et al., 2013).

Glutaraldehyde is commonly used in industry, research labs and, more specific, in hospitals as a disinfectant on dental and medical instruments, such as endoscopes, and surfaces. Glutaraldehyde has a wide range of biocidal activity against both Gram-positive and Gram-negative bacteria, viruses, and spores (Ballantyne and Jordan, 2001; Sehmi et al., 2016). It is a strong cross-linker that combines with multiple molecular functions such as amino and sulfhydryl groups (Okuda et al., 1991). Glutaraldehyde affects cells by binding to nucleic acids and cross-linking enzymes responsible for oxygen uptake, destroying secondary structures and therefore causing disfunction of cytoplasmic molecules and death of cells (Munton and Russell, 1973).

Here, the model bacterium *Escherichia coli* and yeast *Saccharomyces cerevisiae* were used as model organisms to test the impact of autoclaving, microwaving, and glutaraldehyde on the release of DNA from microbial cultures of prokaryotes and eukaryotes, respectively. We elucidated the effects of these three dominant sterilization methods on DNA release, fragmentation, degradation, and amplification capacity on top of cellular inactivation, morphology, and integrity. This incepting work provides first insights to foster the management of the emission of xenogenic pollution.

## Materials and Methods

### Bacterial and Yeast Strains and Culture Preparations

A frozen stock of *Escherichia coli*, DH5α (Cell System Engineering Section, Department of Biotechnology, TU Delft, the Netherlands) was thawed and inoculated into a 300-mL flask containing 200 mL of Luria-Bertani (LB) cultivation broth. This culture was incubated in a rotary shaker for 6 h at 37°C, 200 rpm, where late log phase was reached. A frozen stock of *Saccharomyces cerevisiae*, CEN.PK (Cell System Engineering Section, Department of Biotechnology, TU Delft, The Netherlands) was thawed and inoculated into a 500-mL flask containing 200 mL of yeast extract-peptone-dextrose (YEPD) broth composed of 1% yeast extract, 2% peptone, and 2% glucose/dextrose. This culture was incubated in a rotary shaker for 6 h at 30°C and 200 rpm. Samples of 5 mL at 10^11^CFU L^–1^ of cell cultures were prepared for further sterilization treatments.

### Physical Sterilization Treatment by Microwaving

A microwave oven (Bestron Model ER-M18, 2450 MHz, 230V, 850W) with a rotating table was used. A sealed glass bottle containing 5 mL of each microorganism was placed at the center of the rotating table, 20 cm away from the irradiation source. The microwave was irradiated for a maximum of 30 s within which different time intervals were taken. For each interval time point (0, 5, 10, 12, 14, 16, 18, 20, 25, and 30 s), different sampling tubes were used to ensure that the treatment duration was continuous. Extra exposure times of 40 to 60, 70, and up to 100 s were applied to test for the qPCR-ability of the DNA fragments on top of their release from microbial cells. Microwave was set at maximum power mode in order to avoid its automatic on and off switching. Quality controls were performed with pure 1 ng λ DNA μL^–1^ (Thermo Fisher Scientific Inc., Waltham, MA, United States) by irradiating the sample with microwaves for a maximum of 100 s.

### Chemical Sterilization Treatment by Glutaraldehyde

A generic buffer was prepared by mixing 10 g of sodium bicarbonate (NaHCO_3_) with 90 g of disodium hydrogen phosphate (Na_2_HPO_4_). This buffer mixture was used to adjust the pH of the glutaraldehyde (C_5_H_8_O_2_) solution to an alkaline value of 8.0 which ensures the bactericidal activity of glutaraldehyde (Ballantyne and Jordan, 2001). A 2% (w/w) glutaraldehyde solution (20 g/L, 0.2 M) was prepared and the final pH of the mixture was set at 8.0. Volumes of 12 mL of microorganism suspensions were collected into 15-mL Falcon tubes and were centrifuged at 6000 × *g* at 4°C for 15 min. Afterward, the pellets were resuspended in 12 mL of 1xPBS solution (pH 7.4) and placed in an 18°C water bath for chemical sterilization. Samples were treated with final glutaraldehyde concentrations of 50, 100, 150, 200, 250, and 300 mg L^–1^. Each cell suspension was tested out for 20 min. After reaction, cells were washed and resuspended in 1xPBS solution. The same procedure was followed for quality controls performed with pure λ DNA at 1 ng μL^–1^.

### Thermal Sterilization Treatment by Autoclaving

Sterilization by autoclaving was tested in an autoclave (SHP Steriltechnik AG, Germany) at 110°C and 121°C and 1.1 atm overpressure. The 5 mL cell suspensions were placed in a 25-mL glass tube inside the autoclave and subjected to sterilization under four different autoclaving default programs (program P1: 110°C, 20 min; P2: 110°C, 30 min; P3: 121°C, 20 min; and P4: 121°C, 30 min). Same procedure was followed for quality controls with pure λ DNA at 1 ng μL^–1^.

All sterilization experiments were done in technical triplicates by treating three individual samples taken from each culture.

### Cell Survival

After sterilization, samples of *E. coli* were plated on LB agar plates, and samples of *S. cerevisiae* were plated on YEPD agar plates (100 μL). *E. coli* cells were incubated at 37°C for 24 h, and *S. cerevisiae* cells were incubated at 30°C for 24 h. Plates which contained 30 to 300 colonies were considered suitable for cell counting (Tomasiewicz et al., 1979). Experiments were done in technical triplicates.

### DNA Quantification

After sterilization, 2 mL of each cell sample was centrifuged at 10000 × *g*, 4°C for 3 min. After the first centrifugation, 2 mL of supernatant was collected. To maximize DNA recovery, the residual pellet was washed with 1 mL 1xPBS solution, centrifuged again, prior collecting 1 mL of supernatant. A total of 3 mL supernatant was obtained from each sample. Supernatants and pellets were separated and stored at −80°C pending DNA analysis. Intracellular DNA from the pellets was extracted before and after sterilizations with the DNeasy UltraClean Microbial Kit (Qiagen, Germany). The total DNA content of each sample was the combination of the released DNA obtained on the supernatant fraction plus the DNA obtained from the pellet fraction. The amount of DNA released after sterilization was measured from the supernatant by HS dsDNA Qubit assays (Qubit 3.0, Invitrogen, Carlsbad, CA, United States) according to manufacturer’s protocol.

### DNA Fragmentation

DNA samples were analyzed by gel electrophoresis with agarose at 1% (w/v) (Sigma-Aldrich, Haverhill, United Kingdom) in 1xTAE buffer. DNA was post-stained using SYBR Gold solution (10000x) (Thermo Fisher Scientific, United States) mixed in 1x TAE buffer (AppliChem, Germany) at 1/10K (v/v). Gels after running were immersed into staining buffer for 40 min and were further checked with fluorescence imaging system (Syngene, United Kingdom).

### Primers Selection for *E. coli* and *S. cerevisiae* and Design for λ DNA

Forward and reverse primers were designed to assess the PCR ability of the released DNA fragments after the different sterilization methods used in this study. All primers were purchased from Sigma-Aldrich (Sigma-Aldrich, Haverhill, United Kingdom). The ß-glucuronidase (*uidA*) gene is a molecular marker from *E. coli* that was evaluated by using *uidA* forward (5′-TGGTAATTACCGACGAAAACGGC-3′) and *uidA* reverse (5′-ACGCGTGGTTACAGTCTTGCG-3′) primers (Shimpoh et al., 2017). The TATA binding protein-associated factor (*TAF10*) gene from *S. cerevisiae* was evaluated using *TAF10* forward (5′-ATATTCCAGGATCAGGTCTTCCGTAGC-3′) and *TAF10* reverse (5′-GTAGTCTTCTCATTCTGTTGATGTTGTTGTTG-3′) primers (Masser et al., 2016). The selection of the gene fragments is not random. The ß-glucuronidase (*uidA*) gene has already been reported to efficiently allow for detection and enumeration of *E. coli* while avoiding false positives (Silkie et al., 2008). Regarding *S. cerevisiae*, the TATA binding protein-associated factor TAF10 is a gene, whose expression remains stable independently of growth conditions and strain backgrounds. It makes it a good reference gene for quantitative analysis by qPCR (Teste et al., 2009). For λ DNA, an interesting mobile genetic element was found integrated in its genome: the tyrosine recombinase is involved in the mobility of antibiotic resistance gene cassettes on bacterial class 1 integron-integrase gene (*intI1*). The *intI1* gene was therefore assessed by qPCR. This nicely put this study into the context of horizontal gene transfer phenomena and the emerging concern of antibiotic resistant genes emissions and transfer across the water network. The λ bacteriophage genome was obtained from GenBank (Wu, 1972; Entry Number J02459.1). Primers targeting the λ integrase (*intI1*) gene were designed in house using SnapGene (GSL Biotech)^1^ : λ *int* forward (5′-GTTACCGGGCAACGAGTTGG-3′), λ *int* reverse (5′-ATGCCCGAGAAGATGTTGAGC-3′) primers.

### DNA Extraction and Quantitative PCR (qPCR) Analysis

The quantification of the λ *int* gene, *E. coli uidA* gene, and *S. cerevisiae TAF10* gene in DNA fragments potentially released after sterilization treatments were analyzed by qPCR (QTower 3, Analytica Jena, Germany). For the standard curve construction, genomic DNA from the model organisms *E. coli* and *S. cerevisiae* was isolated using NucleoSpin^®^ Soil (Macherey-Nagel) and Yeast DNA Extraction (Thermo Fisher Scientific Inc., Waltham, MA, United States) kits, respectively. Serial dilutions from 100 ng μL^–1^ down to 10^–5^ ng μL^–1^ were used to generate the standard curve. Validation of the standard curve construction was performed by purchasing 0.3 μg μL^–1^ λ pure DNA (Thermo Fisher Scientific Inc., Waltham, MA, United States). Serial dilution from 1 ng μL^–1^ down to 10^–8^ ng μL^–1^ were used to generate the standard curve. The samples were tested for amplification capacity after sterilization treatments by collecting 1 mL of sterilized culture and centrifugating it at 15000 × *g* for 5 min. The supernatant containing released DNA was collected and diluted 1:10 in ultrapure water (Sigma-Aldrich, Haverhill, United Kingdom) prior being used for qPCR analysis. All qPCR reactions were performed in volumes of 20 μL composed of 10 μL of IQ^TM^ SYBR^®^ Green Supermix (Bio-Rad), 0.2 μL of each primer at 50 μM, 8.6 μL ultrapure water (Sigma-Aldrich, Haverhill, United Kingdom) and 1 μL of template DNA.

The thermal profile selected for the λ *int* gene consisted of 5 min at 95°C hot-start polymerase activation followed by 40 cycles of DNA dissociation at 95°C for 30 s, primers annealing at 55°C for 30 s fragment elongation at 72°C for 30 s, and terminated by holding at 4°C.

The thermal profile selected for the *E. coli uidA* gene consisted of 5 min at 95°C hot-start polymerase activation followed by 40 cycles of DNA dissociation at 95°C for 30 s, primers annealing at 57°C for 30 s, fragment elongation at 72°C for 30 s, and terminated by holding at 4°C.

The thermal profile selected for the *S. cerevisiae TAF10* gene consisted of 5 min at 95°C hot-start polymerase activation followed by 40 cycles of DNA dissociation at 95°C for 30 s, primers annealing at 55°C for 30 s, fragment elongation at 72°C for 30 s, and terminated by holding at 4°C.

### Quality Controls

Different quality controls were used across sterilization experiments and measurements.

Controls for DNA release from cells were produced by bead-milling (or also known as bead-beating) of the bacterial and yeast cultures using the DNeasy UltraClean Microbial Kit. Bead-milled positive controls is considered as an easy and straight-forward method for induced intracellular DNA release (Caleb et al., 2019) and non-bead-milled negative controls were included in the analyses.

Controls for DNA degradation were performed by subjecting free-floating λ DNA to the same sterilization conditions as the cell cultures. It served as a control as it avoids to worry about the effect of cell breakage and other artifacts, and therefore allowed assessing the effect of sterilization on DNA fragmentation and degradation.

### Statistical Analysis

Statistical analyses were performed with R 3.5.1 (R Foundation for Statistical Computing, 2018) and RStudio^2^. For the analysis and determination of the most effective parameters of the sterilization methods effect on DNA amplification a one-way ANOVA test, that can tolerate skewed or kurtotic distribution data, followed by a *post hoc* Tukey’s honestly significant difference (HSD) test at the 0.05 probability level were performed. Figures were prepared using GraphPad Prism 6 (GraphPad Software, San Diego, CA, United States). The absorbance (or “optical density”, OD) of the culture was corrected by assessing the effect of the sterilization methods, assuming homogeneous cultures, thus technical triplicates per biological sample were used.

## Results and Discussion

### Sterilization Methods Inactivated Over 99% of Living Bacterial and Yeast Cells

**FIGURE 1:**
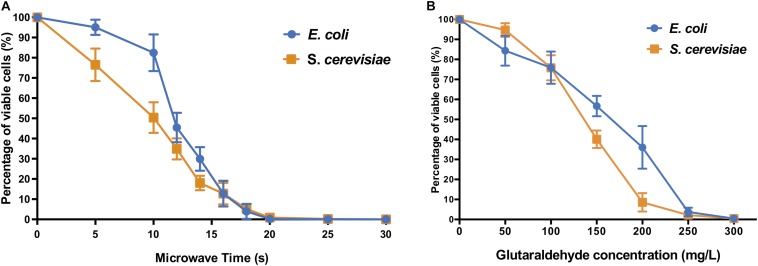
Cell viability of *E. coli* and *S. cerevisiae* after **(A)** different microwave exposure times and **(B)** glutaraldehyde concentrations. The percentage of viable cells is calculated against corresponding control sample cells (untreated with microwave and untreated with glutaraldehyde, respectively).

The microwave effect on cell viability (Figure 1A) showed similar end-points but different profiles for both model prokaryotic and eukaryotic microorganisms. For both *E. coli* and *S. cerevisiae*, the microwaving performance for 15 s was sufficient to reduce the number of cells to nearly 10% of control untreated samples, indicating severe inactivation effect on both microorganisms. Thermal effect is responsible for absorption of microwave energy by cell molecules, producing general heating of the cell (Michaelson, 1974). This causes disarrangement of cell membrane and disruption of cell wall structures by destroying the lipopolysaccharides and peptidoglycan of the cell surface. This results in the emergence of pores, cell aggregations, cytoplasmic proteins aggregation, and changes of membrane permeability (Jankovic et al., 2014; Zeng et al., 2014), explaining why biomolecules such as proteins or nucleic acids (Woo et al., 2000) are detected in the extracellular fraction.

The biocidal effects of the increasing dose of glutaraldehyde were shown on both types of microorganism cells (Figure 1B). *E. coli* and *S. cerevisiae* both displayed significant reduction of cell viabilities compared to their non-treated control samples. When 300 mg L^–1^ glutaraldehyde was applied, both microorganisms decreased to a range between 0.1% to 1% viable cells when compared to control cultures.

The autoclaving treatment was the most effective on both *E. coli* and *S. cerevisiae* (Supplementary Table S1). All autoclaving programs decreased the number of viable cells below the minimum detection limit of <100 CFU mL^–1^, thus resulting in nearly zero survivor cells in the autoclaved cell suspension.

### Cell Lysis and Aggregation After Autoclaving and Microwaving Whereas Loss of Cell Transparency Is Common After All Sterilization Methods

**FIGURE 2:**
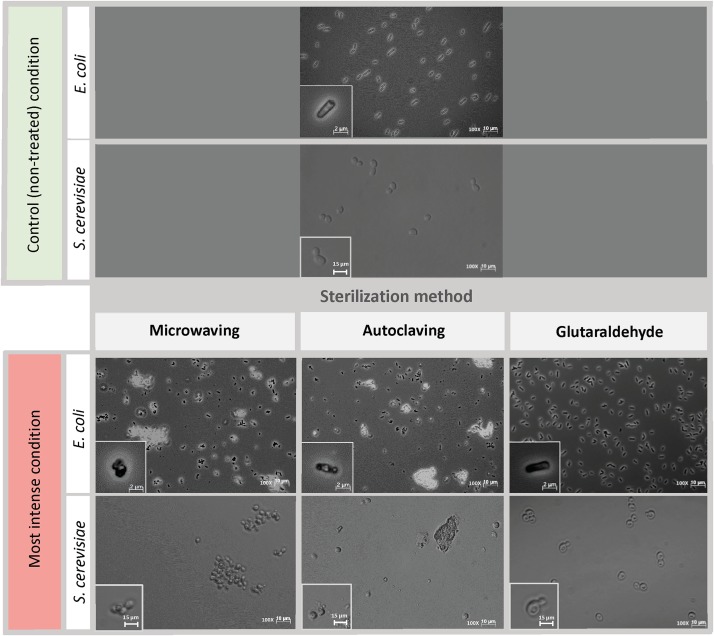
Cell morphology of *E. coli* and *S. cerevisiae* before and after being treated with the harshest sterilization method condition. Pictures were taken at 100x magnification. Upper part: Control (non-treated) condition: microscopic pictures after overnight. Down part: *Left column*: Microwaving effect. Most intense condition corresponds to 30 s microwaves exposure time. *Middle column*: Autoclaving effect. Most intense condition corresponds to P4 (121°C – 30 min). *Right column*: Glutaraldehyde effect. Most intense condition corresponds to 300 mg L^–1^ glutaraldehyde. The morphological structure of single cells at each time point are shown at bottom left of each picture. Same initial control culture was used prior any sterilization treatment.

Phase-contrast microscopy images showed that untreated *E. coli* cells were intact and displayed long rod-shaped structures with smooth surface (Figure 2, upper part). Microwave treatment for 10 s did not affect the overall *E. coli* cell structure as most of the cells maintained their shape (Supplementary Figure S1). After 25 s, cells showed considerable cell debris as well as non-conventional shapes and cell aggregations. Cell transparency was also fully lost after 15 s, becoming dark non-conventional shaped cells compared to the non-treated cells. Short-term microwave treatment preserved cell structure (Supplementary Figures S1E,F) but displayed cell aggregations. The visible damage of *S. cerevisiae* cells emerged 25 s (Supplementary Figures S2D–F), where cell wall destruction resulted in the fusion of *S. cerevisiae* cells into a pool of broken cells. Loss of cell transparency was also observed.

*E. coli* cells displayed severe structural damages under autoclaving treatment as cells no longer preserved the transparent rod-shaped morphology presented in control samples (Figure 2, middle column). Cells were mostly ruptured into pieces of debris, twisted and shrunk, filled with denatured intracellular molecules (Supplementary Figure S3). For *S. cerevisiae*, under the first three types of autoclaving programs, cells completely maintained their spherical shape, and hardly any cell debris were observed (Supplementary Figure S4). Cell metamorphosis occurred under the highest intensity of autoclaving (Figure 2, middle column) where cells lost their clear surface layer and started to perform partial fusions.

The most obvious effect of glutaraldehyde fixation on the morphology of both *E. coli* and *S. cerevisiae* cells (Figure 2, right column) was the change of cell transparency. The intracellular area of *E. coli* cells turned black when fixed with glutaraldehyde and for *S. cerevisiae*, dark spots were present in their cytoplasm. No cell aggregation nor significant cell damage was observed. Cells from both microorganisms still preserved their structural frame even when the highest concentration of glutaraldehyde of 300 mg L^–1^ was applied (Supplementary Figures S5, S6).

### Significant DNA Release Observed Under Microwave and Autoclaving Sterilizations

**FIGURE 3:**
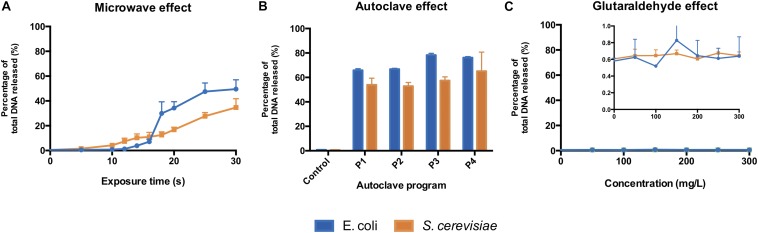
Total DNA released from *E. coli* and *S. cerevisiae* treated with **(A)** different microwave exposure times, **(B)** different autoclave programs and **(C)** different glutaraldehyde concentrations. The percentage shows the ratios of the amount of DNA (dsDNA) released in the supernatant against the total amount of DNA (released and remained combined). Autoclave programs: P1 (110°C – 20 min), P2 (110°C – 30 min), P3 (121°C – 20 min) and P4 (121°C – 30 min). Control (non-treated cells). 100% correspond to 59.1 ng μL^–1^ and 73.9 ng μL^–1^ for *E. coli* and *S. cerevisiae*, respectively.

The microwave effect on the total DNA released by *E. coli* was observed after 12 s and increased abruptly after 16 s of exposure (Figure 3A). In *S. cerevisiae* cultures, the total DNA released increased constantly from the beginning of the treatment (Figure 3A). Even if *S. cerevisiae* cells released DNA constantly from the start, their cell wall protective ability was higher than the *E. coli* ones: yeasts displayed higher resistance in terms of cell structural collapse and DNA release into the extracellular media. This links to the microscopic pictures presented in Figure 2, where after 20 s under the microwaves, the *S. cerevisiae* cells preserved their structure whereas the *E. coli* cells lysed and aggregated (Supplementary Figures S1, S2). Resistance differences also resulted in a lower percentage of total DNA released from *S. cerevisiae* at maximum exposure (30 s) of microwave sterilization. Nearly 35% of total DNA (73.9 ng μL^–1^) was released from *S. cerevisiae* cells at 30 s, in contrast to almost 50% (59.1 ng μL^–1^) from *E. coli* (Supplementary Figures S7A–D).

All types of autoclaving led to considerable amount of DNA released to the medium from both microorganisms (Figure 3B). Different autoclaving programs caused 65% (P1) to nearly 80% (P4) of total DNA leakage from *E. coli* cells while lower amounts of total DNA, 53% (P1) to 65% (P4), were observed on *S. cerevisiae* cultures (Supplementary Figure S8).

When an increasing amount of glutaraldehyde was applied from 0 to 300 mg L^–1^, the percentage of total DNA released to extracellular medium (Figure 3C) stayed either steady at 0.6% for *S. cerevisiae* or fluctuating from 0.4 to 0.9% for *E. coli*. Almost all the DNA from both microorganisms remained intracellularly (Supplementary Figures S7E–H). Glutaraldehyde displays a protective effect against cell lysis (Azeredo et al., 1999; D’Souza and Marolia, 1999) and strongly inhibits autolytic and proteolytic processes (Flores et al., 1996). This explains why DNA was not released into the extracellular fraction. Glutaraldehyde does perform cross-linking reactions with compounds present in the cell outer layers, thus negatively altering the permeability and transportability of cell membranes (Munton and Russell, 1973; Maillard, 2002; Sehmi et al., 2016). Our observations show that this chemical exposure time (20 min) is enough to inactivate cells and prevent the transport and release of nucleic acids across membranes.

### Long Microwave Exposures and All the Autoclave Programs Showed Severe DNA Damage

The DNA fragments of the untreated *E. coli* control sample displayed on agarose gel have a size above 10 kb. When cells were treated with increasing duration of microwave, the intracellular DNA bands were less intense and displayed a slight decline gradient of DNA sizes, trailed by smears at different degrees (Supplementary Figure S9A). For *S. cerevisiae* (Supplementary Figures S9C,D), the DNA extracted from untreated cells showed multiple bands of various lengths from approximately 400 bp to over 10 kb. After 25 s of microwaving, no clear bands but smears were displayed on the gels. An exposure of 30 s resulted in elimination of *S. cerevisiae* visible bands in the intracellular fraction (Supplementary Figure S9C). The fragmentation patterns in *E. coli* matched the measurements of DNA content (Figure 3A) for which a significant amount of DNA was released from 18 s onward (Supplementary Figure S9B). An increasing gradient of the free-floating DNA intensity on the gel from *S. cerevisiae* (Supplementary Figure S9D) fitted to the gradual DNA release (Figure 3A).

In contrast to microwaving, extracellular and intracellular DNAs from both microorganisms were more highly fragmented and/or degraded when heat and pressure (1.1 atm overpressure) were applied (Figure 3B), displaying smears on agarose gels (Supplementary Figures S10A–D). The highest autoclaving program (121 °C, 30 min) showed the strongest DNA damage and release when compared with controls and other conditions (Supplementary Figures S5A–C, lanes 5). Even when the harshest autoclaving was applied, intense bands of DNA were still observed on the agarose gels. The degree of intracellular DNA degradation was lower than the released DNA after autoclaving treatment, presumably due to the protection of cells on its cytoplasmic DNA against external damage (Espy et al., 2002; Elhafi et al., 2004; Simmon et al., 2004). A possible reason why *E. coli* shows higher resistance to stress when compared to *S. cerevisiae*, apart from their higher surface to volume ratio, could be its polyunsaturated fatty acids (PUFAs) composition in their membrane: they contribute to cell membrane flexibility. PUFAs level in *S. cerevisiae* membranes are low or inexistent when growing under normal conditions (Kajiwara et al., 1996; Uemura, 2012) whereas in *E. coli* cells are higher (Shokri and Larsson, 2004).

Intracellular DNA of *E. coli* treated with the highest concentration of glutaraldehyde resulted in a smear with high fragment lengths (Supplementary Figure S11A, lane 7). Intracellular genomic DNA on *S. cerevisiae* did not result in the absence of DNA bands but a decrease of the band intensity (Supplementary Figure S11C, lane 7). The extracellular DNA from both types of microorganisms (Supplementary Figures S11B–D) showed similar patterns containing short DNA fragments from <100 bp to >200 bp, same as their corresponding untreated controls. The presence of residual extracellular DNA primarily results from the natural DNA release of microbial cells even before application of glutaraldehyde (Kloos et al., 1994).

### Autoclaving Was the Most Effective in Compromising PCR-Ability

The amplification efficiency of selected genes was assessed after sterilization by qPCR. Differences of log_10_ number of DNA copies per mL between bead-milled control samples (autoclaving and microwaving) or non-bead-milled control samples (glutaraldehyde) and treated samples gave an insight on the DNA integrity after sterilization. For autoclaving and microwaving, log_10_ values close to the control indicated a high number of amplifiable DNA sequences available in the sample. This meant that DNA was not degraded enough and maintained its integrity. For glutaraldehyde, values close to the non-bead-milled samples indicated no DNA release nor an effect on PCR ability. Glutaraldehyde treatment did not release any significant amount of DNA in the supernatant (Figure 3C). For this reason, a non-bead-milled control was added. It was important to know if the amount of DNA released after glutaraldehyde treatment was similar to non-sterilized samples, where culture supernatant was basically assessed by qPCR without being sterilized. This gave an idea about the number of DNA copies available that could have been released by some passive release mechanisms or some basal cells decay during the overnight culture.

Bead-milling was the method of choice to release most of the intracellular DNA and is conventionally used for DNA extractions from microbial samples, but sometimes it is possible that the sterilization experiments showed higher release. It was expected with the mechanical disruption that DNA was released (but potentially not denatured). With the sterilizations here applied, DNA was expected to be released and potentially denatured and thus less PCR-able. Controls are not unique for all the cases: controls were included simultaneously to sterilized samples.

For qPCR analysis, the amplified DNA fragments should not exceed 200–250 bp. This is a relatively short DNA fragment size. It was hypothesized that qPCR measurements will highlight whether the DNA released after sterilization was strongly damaged. A high fragmentation of genomic DNA would result in DNA fragment sizes below 200–250 bp, allowing to see an effect in loss of PCR ability.

**FIGURE 4:**
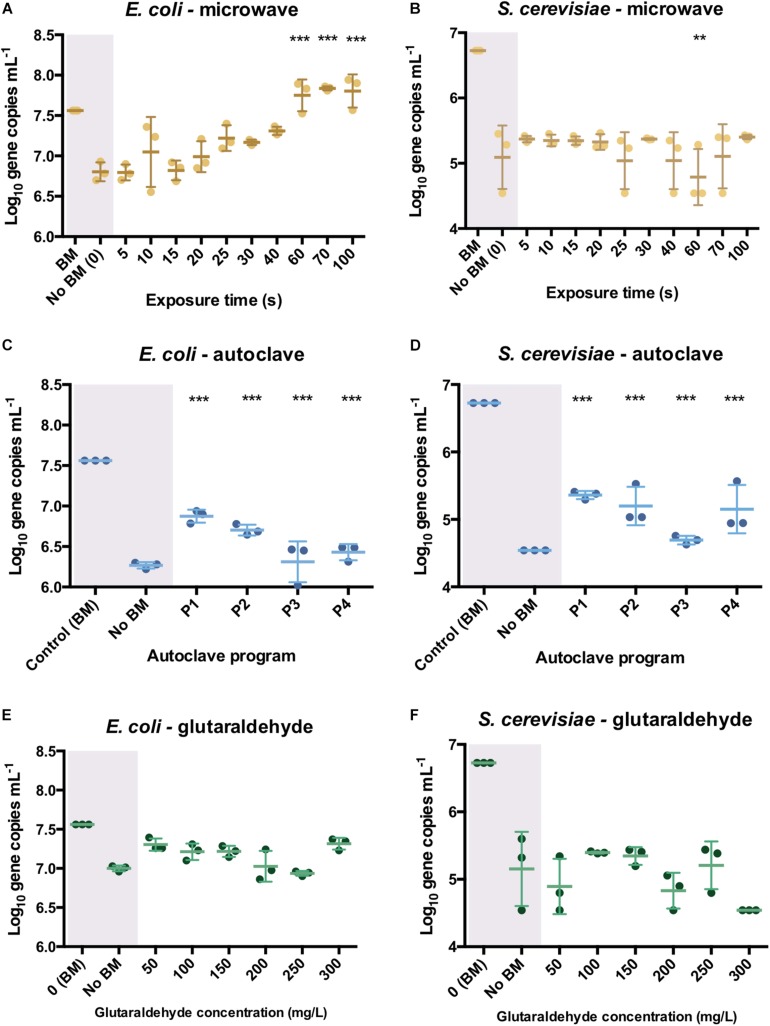
qPCR results after the different sterilization methods. Number of DNA copies obtained on supernatant of pure *E. coli* and *S. cerevisiae* cultures after microwave **(A,B)**, autoclave **(C,D)** and glutaraldehyde **(E,F)** treatments. The results are expressed in log_10_ copies per mL. Autoclave programs: P1 (110°C – 20 min), P2 (110°C – 30 min), P3 (121°C – 20 min) and P4 (121°C – 30 min). BM: Bead-Mill treatment. ANOVA followed by *post hoc* Tukey test, **p < 0.05, ***p < 0.005.

In *E. coli* cultures, no effect on PCR ability was observed when DNA was released from cells after microwaving. There were higher initial (5 s) log_10_ differences with the bead-milled control sample (0.77 ± 0.02) values due to lack of DNA available on the extracellular fraction (6.79 log_10_ gene copies mL^–1^). DNA was exponentially released after 25 s (7.22 ± 0.07 log_10_ gene copies mL^–1^, Figure 4A), and significantly released after 70 s (7.84 ± 0.01 log_10_ gene copies mL^–1^). After 100 s exposure, DNA was released from cells (7.81 ± 0.08 log_10_ DNA copies) as its number of sequences even got higher than the control values (7.56 ± 0.01 log_10_ DNA copies) but its PCR ability was not compromised. Regarding autoclaving, a signal was observed even after P3 and P4 programs were applied (Figure 4C): 1.28 ± 0.11 and 1.16 ± 0.04 log_10_ gene copies per mL difference with the bead-milled control, respectively. Glutaraldehyde did not have a significant effect on the PCR ability (Figure 4E) mainly because samples treated with glutaraldehyde did not release DNA (Figure 3C). In this case, the non-bead-milled control contained a relatively high number of gene copies in the supernatant, which anyway corresponded to similar values when different glutaraldehyde concentrations were applied. Overall, the qPCR background was relatively high for both the controls and the samples (Figure 4E). An analysis of variance (ANOVA) on these scores yielded significant variation among autoclaving and microwaving treatments but not glutaraldehyde treatments. When compared with the bead-milled control sample, *F* = 10245.62 and *F* = 149.09, *p* < 0.0001 were observed for autoclaving and microwaving, respectively (Supplementary Table S2). A *post hoc* Tukey test showed that all the autoclaving treatments (P1, P2, P3 and P4) and control belonging group differed significantly at *p* < 0.05 (table not shown) on the PCR-ability of release DNA from *E. coli* cultures. Regarding microwaving, the Tukey test showed significant (*p* < 0.05) differences from 60 to 100 s exposure times.

In *S. cerevisiae* cultures, DNA was released in a constant trend when microwaving meaning that lower amounts of DNA were available in the supernatant per time unit. This balance of DNA release seems to be proportional to degradation over time. This was reflected in Figure 4B, where constant values around 1.5 log_10_ differences are observed over time. A decrease of 4.78 ± 0.18 log_10_ DNA copies per mL at 60 s was observed but went up to 5.11 ± 0.2 log_10_ DNA copies per mL at 70 s. After the longest microwaving exposure time of 100 s, the number of amplicons increased (5.4 ± 0.02 log_10_) indicating no significant effect on DNA integrity (i.e., can be amplified by qPCR). DNA release pattern and PCR ability from *S. cerevisiae* differ drastically from the exponential released observed in *E. coli* cultures (Figure 4A). After autoclaving, qPCR still detected some sequences (1.62 ± 0.15 log_10_ difference) even after the most intensive programs (P4: 121°C, 30 min, Figure 4D). However, P3 (121°C, 20 min) resulted in lower amplifiable DNA sequences (2.08 ± 0.03 log_10_ difference). As observed in *E. coli* cultures, glutaraldehyde did not have a significant effect on DNA PCR ability mainly because samples released non-detectable levels of DNA (Figure 4E). It was observed that DNA found in supernatant after glutaraldehyde treatment was at a similar level to the negative control, confirming that DNA was not released and thus, not amplified (Figure 4F). An ANOVA on these scores yielded significant variation among autoclaving treatment but not after microwave and glutaraldehyde treatments. When compared with the bead-milled control sample, *F* = 137.77, *p* < 0.0001 were observed for autoclaving (Supplementary Table S2). Tukey test showed that all the autoclaving treatments (P1, P2, P3, and P4) and control belonging group differed significantly at *p* < 0.05 (table not shown) on the PCR-ability of release DNA from *S. cerevisiae* cultures. Regarding microwaving and glutaraldehyde, no significant (*p* > 0.05) mean differences were observed supporting the low effect of these methods on PCR ability. High temperature in combination with high pressure massively degrades DNA even in a short period of time of 5 min (Karni et al., 2013).

Some studies have shown that dry autoclaving at 100°C for 10 min has been already sufficient to result in not amplifiable DNA from soybean (Bergerová et al., 2011). However, genomic DNA amplification has been observed at different autoclaving times (from 10 to 40 min) out of cultures of *Pseudomonas aeruginosa, Salmonella Nottingham*, and *Escherichia coli*. Collectively, this suggests a potential risk made by residual genomic DNA after inactivation of microbial cells due to potential horizontal gene transfer phenomena (Yap et al., 2013). Little is known on the amplification capacity of free DNA after autoclaving treatment of industrial model organisms, being a source of xenogenic contamination out of industries and laboratories.

DNA fragmentation was experienced with both autoclaving and microwaving. Although fragmented, the DNA pieces could still be amplified to some extent by qPCR. The sterilization exposure time was sufficient to break the cells, to release and to fragment, while not sufficiently long to lead to a DNA residue degraded enough to affect its PCR ability. For autoclaving and microwaving we can postulate the following mechanistic steps in the sterilization process: (*i*) cells breakage, (*ii*) DNA release, (*iii*) DNA fragmentation, (*iv*) DNA degradation.

### Quality Test Experiments Using Autoclave and Microwave Treatments Showed Faster Pure DNA Degradation Patterns When Compared to Pure Cultures

The different sterilization treatments were tested out *in vitro* on pure phage λ DNA and analyzed by gel electrophoresis to assess afterward their effect on free extracellular DNA.

**FIGURE 5:**
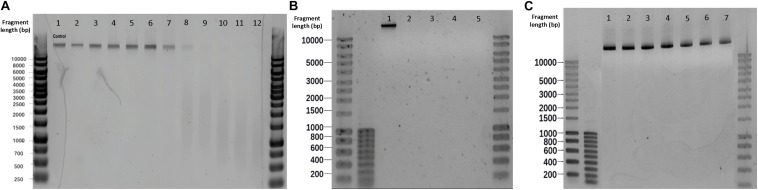
Effect of different sterilization methods on λ DNA fragmentation. **(A)** Microwaving. Lanes 1–12 correspond to 0, 5, 10, 15, 20, 25, 30, 40, 50, 60, 70, 100 s microwave exposure time, respectively. **(B)** Autoclaving. Lanes 1–5 correspond to control, P1, P2, P3, and P4, respectively. Autoclave programs: P1 (110°C – 20 min), P2 (110°C – 30 min), P3 (121°C – 20 min) and P4 (121°C – 30 min). **(C)** Glutaraldehyde. Lanes 1–7 correspond to 0,50, 100, 150, 200, 250 and 300 mg L^–1^ glutaraldehyde, respectively.

All the autoclaving programs applied on pure λ DNA reflected neither visible bands nor smears on gel electrophoreses, meaning that complete degradations of naked λ DNA occurred (Figure 5B). DNA was not visible for SYBR-Gold staining. In contrast with the DNA treated by autoclaving *in vivo* (Supplementary Figure S10), where intracellular DNA migrated slower and displayed higher fragment length than released DNA. The disappearing of λ DNA treated with high temperature and pressure manifested the protection of DNA by cells against damage. Slight increased pressure and temperatures decay the primary structure of double-strand DNA by hydrolyzing its chemical bonds (Masters et al., 1998). This significantly affects the DNA stability and causes DNA fragmentation. This is remarkable with free DNA and was confirmed by the autoclaving of pure free λ DNA, where gels did exhibit severe DNA fragmentation (Figure 5B).

An exposure of 30 s to microwaves was not sufficient to cause degradation and fragmentation to free pure λ DNA: the band obtained after treatment (Figure 5A. Lane 7) remained identical to the untreated template (Figure 5A. Lane 1). At 40 s, the DNA band lost its intensity, resulting in a smear with lower fragment lengths. After 60 s, the band disappeared and completely turned into a smear. In comparison with the results in microbial cultures (Supplementary Figure S9), the phage λ DNA treated with the same duration of microwaving resulted in slower migrations across the gel than the DNA released from *E. coli* and *S. cerevisiae* cultures. Non-thermal factors are all of the effects that are not caused by an increase of temperature, especially when low frequencies and intensities are applied (Belyaev et al., 1992). Belyaev (2005) has shown that radiation-induced DNA breaks could not be repaired after non-thermal microwave exposure. This effect inhibits DNA repair being a plausible cause for cells inactivation. When moving from the cellular to the DNA level, microwaves can destroy DNA by denaturation, degradation, and fragmentation (Fang et al., 2011; Yang and Hang, 2013).

No DNA degradation nor fragmentation could be observed when increasing glutaraldehyde concentrations (Figure 5C).

### Pure λ DNA Amplification Efficiency Decreased Under Long Microwave Exposures and All Autoclaving Programs

The qPCR of the pure bacteriophage λ *int* gene was used to evaluate the extracellular DNA capacity to be amplified right after the different sterilization methods.

**FIGURE 6:**
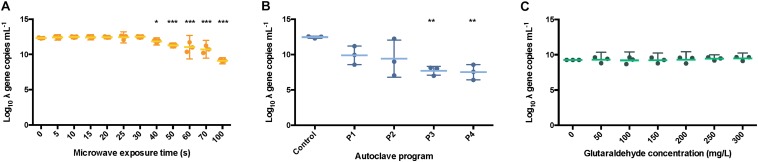
Number of *int* copies from λ DNA available after microwaving **(A)**, autoclaving **(B)** and treating with glutaraldehyde **(C)**. The results are expressed in log_10_ copies per mL. The middle line represents the mean, and the whiskers represent the 95% CI. Autoclave programs: P1 (110°C – 20 min), P2 (110°C – 30 min), P3 (121°C – 20 min) and P4 (121°C – 30 min). Control: non-sterilized samples corresponding to 1 g L^–1^ λ DNA.

All the autoclave treatments were shown to significantly affect the PCR ability of the λ DNA (Figure 6B) notably when applying program P4 (121 °C, 30 min) when compared with the non-treated λ DNA control. Differences of 2.56 ± 0.61, 3.04 ± 1.22, 4.75 ± 0.24, 4.96 ± 0.28 logs were observed for P1, P2, P3 and P4 versus the control, respectively. An ANOVA on these scores yielded significant variation among autoclaving treatments when compared with the control, *F* = 11.41, *p* < 0.001 (Supplementary Table S3). A Tukey test showed that all the autoclaving treatments (P1, P2, P3 and P4) and control belonging group differed significantly at *p* < 0.05 (table not shown) on the PCR-ability of λ DNA.

It took around 30 to 50 s to detect high concentrations of DNA in the extracellular fraction and around more than 100 s to start seeing a decay in PCR ability. From the combination of experiments with microbial cultures and free-floating λ DNA, exposure time longer than 100 s would be necessary to inactivate cells, release DNA, and fragment it. Otherwise, no effect will be observed on DNA integrity.

Similar results were observed when samples were exposed for over 40 s, 0.5 ± 0.09 logs difference, under microwaves (Figure 6A). Significant variation among microwaving treatments when compared with the control, *F* = 26.04, *p* < 0.0001 (Supplementary Table S4) was observed. Moreover, a Tukey test showed that exposure times over 60 s, 1.31 ± 0.17 logs difference, differed significantly at *p* < 0.05 (table not shown). This is supporting the clear band loss after 60 s during gel electrophoresis (Figure 5A). At 100 s, a difference of 3.23 ± 0.06 logs with the untreated control sample was observed.

Under microwave treatment, DNA damage on pure λ DNA was more severe than in DNA released from microbial cells. Although the DNA of *E. coli* (approximately 4 Mb) and *S. cerevisiae* (approximately 12.1 Mb) harbors significant differences in the size of their genomes, which are also both larger than the size of λ DNA (48 kb) (Blattner et al., 1997; Engel et al., 2014), the main reason resided in the contact time with the sterilization treatments.

The sterilization processes by microwaving and autoclaving conducted on cells were described to involve 4 different steps from cell breakage to DNA release, DNA fragmentation, and DNA degradation. In most of experiments performed on cell cultures, the contact time was not long enough to enable degradation of DNA beyond its release. In the case of the free DNA control, λ DNA was directly exposed from start to the sterilization treatments. λ DNA was therefore longer in contact with the sterilization conditions than the DNA released from cell. This longer exposition led to degradation of λ DNA to some extent.

In addition, when cells are treated with microwaves, temperature in highly concentrated cell suspensions increased faster than in low cell concentration suspensions (Zeng et al., 2014). Hence, the DNA released from suspensions of *E. coli* and *S. cerevisiae* could end up with a higher temperature compared to the pure λ DNA solution when treated with the same duration of microwave, accelerating DNA degradation. However, even if DNA in suspension could end up with higher temperatures, first it should be released before being nakedly exposed to the environmental conditions of the system.

In our study, a first question targeted whether DNA was released: this was confirmed by Qubit results (Figure 3A) and gel electrophoresis (Supplementary Figure S9). A second question targeted whether the integrity of DNA was impacted during the treatments, which was checked by qPCR. The summary of the microwave effect on microbial cultures (Figure 3A; Figures 4A,B) and on naked pure DNA (Figures 5A, 6A) does provide important information for bench-top practice if microwave is the desired method to sterilize your microbial culture. Our results suggest that an exposure time of more than 100 s is needed under our experimental conditions to efficiently inactivate microorganisms and degrade the potentially harmful DNA they may contain. The initial 40–50 s are needed to inactivate/break cells and release the DNA out of the cells, plus another extra time to observe a degradation of the DNA and decay of its PCR ability (i.e., its potential to be biologically re-used and genomically integrated by microorganisms).

In contrast, the glutaraldehyde treatments did not affect the amplification capacity of pure λ DNA (Figure 6C). An ANOVA on these scores did not yield significant variation among the different concentrations of glutaraldehyde when compared with the control (*F* = 0.70, *p* > 0.658, Supplementary Table S4), supporting the results obtained from the DNA fragmentation experiments (Figure 5C). No significant effect on amplification ability was observed when a standard incubation time of 20 min was applied. Glutaraldehyde damages DNA (Hopwood, 1975) and compromises the PCR ability of DNA after some days of incubation only (Das et al., 2014). In clinical procedures, an exposure of 20 min at 20°C in a 2% w/w glutaraldehyde solution is solely used to disinfect medical instruments (Park et al., 2013). We showed that this short incubation time of 20 min was not enough to impact the integrity and PCR ability of DNA. Overall, glutaraldehyde offers an efficient way to disinfect and contain DNA and xenogenic elements inside cells.

### Outlook

Overall, all common sterilization methods here tested are effective to inactivate microorganisms, highlighting short incubation time of 20 min with glutaraldehyde for its capacity to avoid DNA release. In terms of DNA loss of integrity, autoclave is shown to be the most effective method. However, integrity of released DNA is not completely compromised as shown by qPCR results. This opens a window for improvement in case total extracellular DNA degradation was desired. Alternatives to standard procedures are combination of methods here tested or further steps toward total removal such as ethylene oxide that has been shown to reduce DNA amplification when long exposure times are applied (Shaw et al., 2008). Fragmented sequences as short as 20 bp have been shown to be taken up and incorporated into the bacterial DNA, including mammoth DNA (Overballe-Petersen et al., 2013). Even if the majority of short residual DNA fragments will be re-metabolized in case they are taken up, there is a probability to be genome integrated generating new diversity (Overballe-Petersen and Willerslev, 2014).

Horizontal gene transfer phenomena from sterilized cultures may exchange all kind of DNA fragments as soon as these enter microbiome hotspots such as wastewater treatment plants (Miller et al., 2016). New microbial diversity can be generated through gene transfer, but also undesirable fragments such as ARGs and pathogenic islands could be unfavorably mobilized by microorganisms (Jørgensen et al., 2014; Messerer et al., 2017). This underlies the ubiquity and potentiality of these DNA fragments generated after sterilizations. Further research on the quality and composition of released DNA as well as rates of horizontal gene transfer are necessary to develop risk assessments strategies and to address the impact of the standard sterilization methods on biosafety and environmental and public health.

## Data Availability Statement

All datasets generated for this study are included in the article/Supplementary Material.

## Author Contributions

DW, BA, and DC-F designed the study with inputs of QL and ML. DC-F and QL performed the experimental investigations. DC-F and DW wrote the manuscript with direct contribution, edits, and critical feedback by all authors.

## Conflict of Interest

The authors declare that the research was conducted in the absence of any commercial or financial relationships that could be construed as a potential conflict of interest.

## Footnotes

1 www.snapgene.com

2 https://www.rstudio.com/
