# Supplementary material for: Anticipating Xenogenic Pollution at the Source: Impact of Sterilizations on DNA Release From Microbial Cultures
Source: Front Bioeng Biotechnol. 2020 Mar 13;8:171. doi: 10.3389/fbioe.2020.00171 (PMC7082761; doi:10.3389/fbioe.2020.00171)
Supplement: Supplementary file 1 [file Data_Sheet_1.pdf]

Supplementary information to:

Anticipating xenogenic pollution at the source: Impact of sterilizations on DNA release from microbial cultures

David Calderón-Franco, Qingnan Lin, Mark C. M. van Loosdrecht, Ben Abbas, David G. Weissbrodt*

Department of Biotechnology, Delft University of Technology, van der Maasweg 9, 2629 HZ Delft, The Netherlands

***Correspondence:** Prof. David Weissbrodt, Assistant Professor, Weissbrodt Group for Environmental Life Science Engineering, Environmental Biotechnology Section, Department of Biotechnology, Faculty of Applied Sciences, TU Delft, van der Maasweg 9, Building 58, 2629 HZ Delft, the Netherlands, Tel: +31 15 27 81169 ; E-mail: d.g.weissbrodt@tudelft.nl

Table S1. *E. coli* and *S. cerevisiae* cell viability after different autoclaving programs. The percentage of viable cells is calculated against corresponding control sample cells (non-autoclaved cells).

	E. coli		*S. cerevisiae*	
Treatment condition	Cell number Log (CFU) mL^{-1}	Viability (%)	Cell number Viability Log (CFU) mL^{-1} (%)	
**Autoclaving program**				
110 °C, 20 min	*NA	*NA	*NA	*NA
110 °C, 30 min	*NA	*NA	*NA	*NA
121 °C, 20 min	*NA	*NA	*NA	*NA
121 °C, 30 min	*NA	*NA	*NA	*NA

*NA (not applicable): Cell concentration reduced under the accurate detection limit of 100 CFU mL^{-1}.

Table S2. Fixed-Effects ANOVA results using the different methods Log_{10} *uidA* copies mL^{-1} after sterilization methods on *E. coli* as the criterion

Treatment condition	***Microwaving***						
Predictor	Sum of Squares	df	Mean Square	F	p	partial η2	partial η2 90% CI [LL, UL]
(Intercept)	149.09	1	149.09	4795.50	.000		
Exposure time (s)	5.33	11	0.48	15.57	**.000** ***	.88	[.69 .88]
Error	0.75	24	0.03				

Treatment condition	***Autoclaving***						
Predictor	Sum of Squares	df	Mean Square	F	p	partial η2	partial η2 90% CI [LL, UL]
(Intercept)	172.84	1	172.84	10246.62	.000		
Autoclave program	3.04	4	0.76	45.10	**.000** ***	.95	[.81, .96]
Error	0.17	10	0.02				

Treatment condition	***Glutaraldehyde***						
Predictor	Sum of Squares	df	Mean Square	F	p	partial η2	partial η2 90% CI [LL, UL]
(Intercept)	156.07	1	156.07	6266.22	0.000		
Glutaraldehyde concentration (mg L^{-1})	0.23	6	0.04	1.53	.240	.40	[.00, .46]
Error	0.35	14	0.02				

Note. LL and UL represent the lower-limit and upper-limit of the partial η2 confidence interval, respectively.

Table S3. Fixed-Effects ANOVA results using the different methods Log_{10} *TAF10* copies mL^{-1} after sterilization methods on *S. cerevisiae* as the criterion

Treatment condition			***Microwaving***				
Predictor	Sum of Squares	df	Mean Square	F	p	partial η2	partial η2 90% CI [LL, UL]
(Intercept)	85.85	1	85.85	960.59	.000		
Exposure time (s)	3.88	11	0.35	3.95	**.002** **	.64	[.21, .65]
Error	2.15	24	0.09				

Treatment condition			***Autoclaving***				
Predictor	Sum of Squares	df	Mean Square	F	p	partial η2	partial η2 90% CI [LL, UL]
(Intercept)	137.77	1	137.77	2660.87	.000		
Autoclave program	7.48	4	1.87	36.10	**.000** ***	.94	[.77, .95]
Error	0.52	10	0.05				

Treatment condition			***Glutaraldehyde***				
Predictor	Sum of Squares	df	Mean Square	F	p	partial η2	partial η2 90% CI [LL, UL]
(Intercept)	87.39	1	87.39	666.34	.000		
Glutaraldehyde concentration (mg L^{-1})	0.81	6	0.14	1.04	.447	.31	[.00, .36]
Error	1.84	14	0.13				

Note. LL and UL represent the lower-limit and upper-limit of the partial η2 confidence interval, respectively.

Table S4. Fixed-Effects ANOVA results using the different methods Log_{10} λ *int* gene copies mL^{-1} after sterilization methods as the criterion

Treatment condition	***Autoclaving***						
Predictor	Sum of Squares	df	Mean Square	F	p	partial η2	partial η2 90% CI [LL, UL]
(Intercept)	466.00	1	466.00	228.31	0.000		
Autoclave program	48.25	4	12.06	5.91	**0.01** **	.70	[.17, .76]
Error	20.41	10	2.04				

Treatment condition	***Microwaving***						
Predictor	Sum of Squares	df	Mean Square	F	p	partial η2	partial η2 90% CI [LL, UL]
(Intercept)	456.43	1	456.43	5556.42	0.000		
Exposure time (s)	36.32	11	3.30	40.20	**.000** ***	.95	[.87, .95]
Error	1.92	24	0.08				

Treatment condition	***Glutaraldehyde***						
Predictor	Sum of Squares	df	Mean Square	F	p	partial η2	partial η2 90% CI [LL, UL]
(Intercept)	257.46	1	257.46	1925.41	0.000		
Glutaraldehyde concentration (mg L^{-1})	0.19	6	0.03	0.24	.956 -	.0	[.00, 1.00]
Error	1.87	14	0.13				

Note. LL and UL represent the lower-limit and upper-limit of the partial η2 confidence interval, respectively.

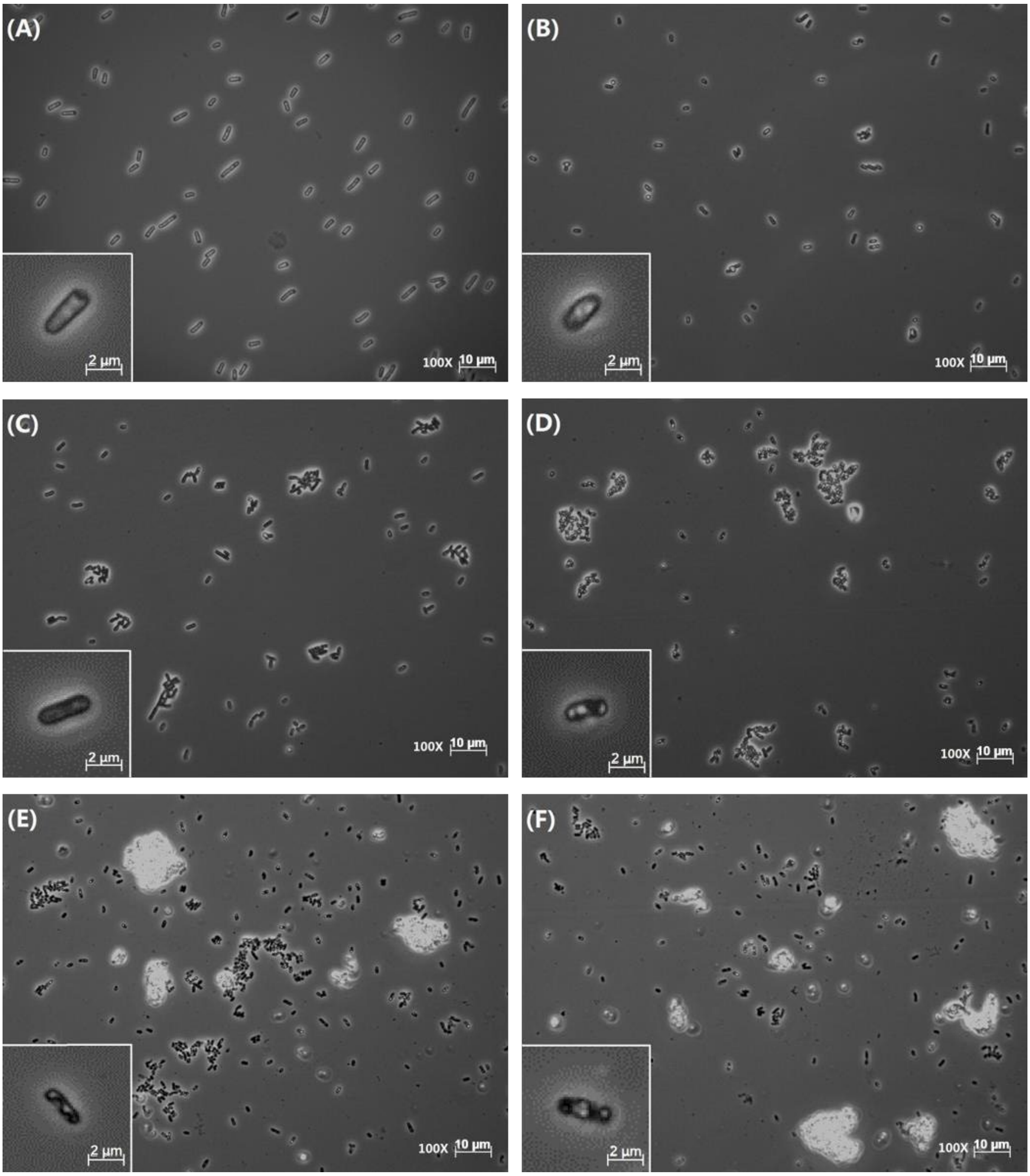

Figure S1. Microscopic pictures of *E. coli* cells treated with microwave (2450 MHz, 230V, 850W) sterilization method at 10 s **(b)**, 15 s **(c)**, 20 s **(d)**, 25 s **(e),** and 30 s **(f)** in comparison with 0 s untreated control cells **(a)**. The morphological structure of single cells at each time point are shown at bottom left.

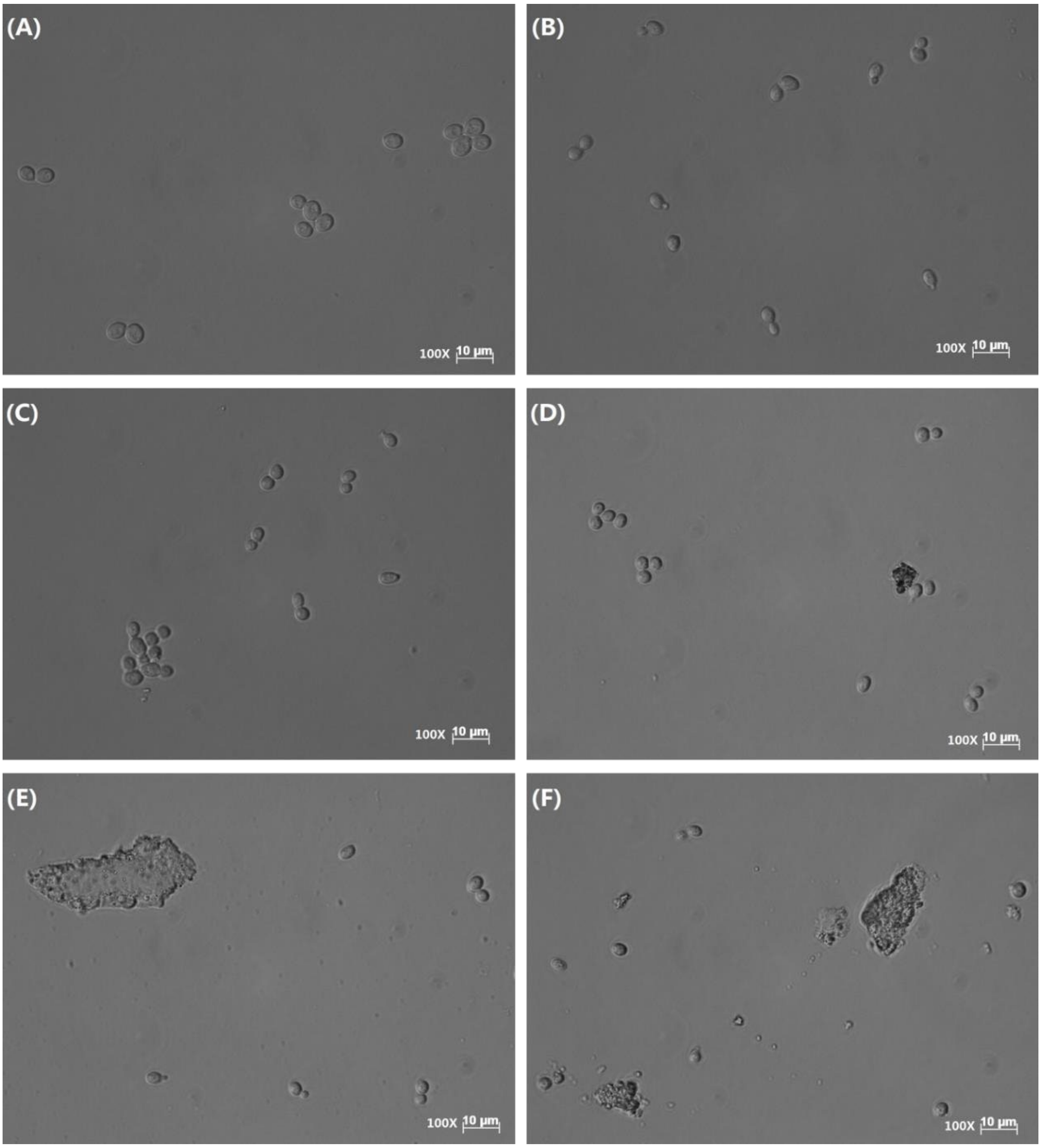

Figure S2. Microscopic pictures of *S. cerevisiae* cells treated with microwave (2450 MHz, 230V, 850W) sterilization method at 10 s **(b)**, 15 s **(c)**, 20 s **(d)**, 25 s **(e),** and 30 s **(f)** in comparison with 0 s untreated control cells **(a)**.

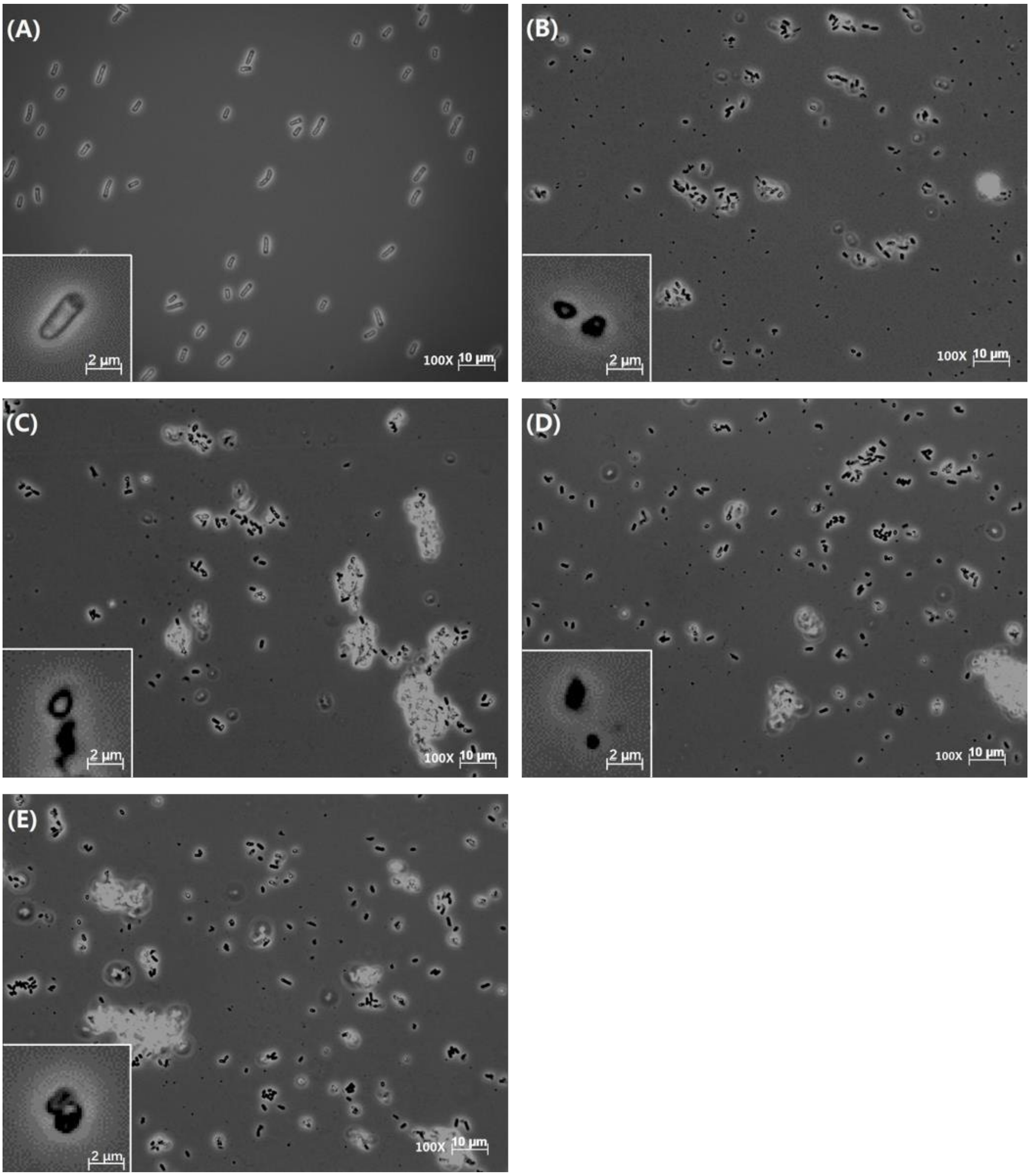

Figure S3. Microscopic image of *E. coli* cells through different type of autoclaving program. Cells after autoclaving program of 110 °C, 20 min (**b**), 110 °C, 30 min (**c**), 121 °C, 20 min (**d**), 121°C, 30 min (**e**) are in comparison with untreated control cells (**a**). The morphological structure of single cells under each types of autoclaving program are shown at bottom left.

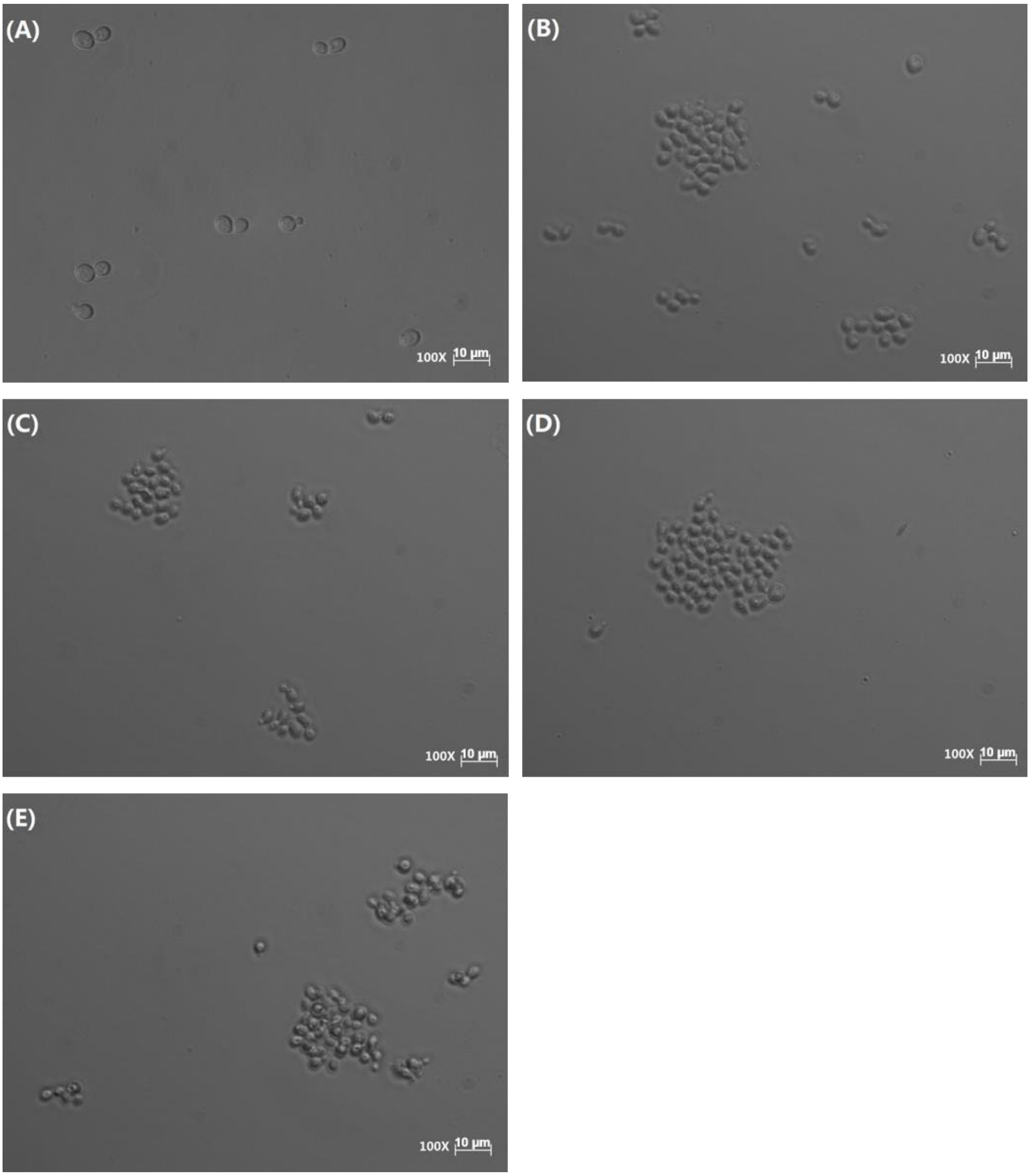

Figure S4. Microscopic image of *S. cerevisiae* cells through different type of autoclaving program. Cells after autoclaving program of 110 °C, 20 min (**b**), 110 °C, 30 min (**c**), 121 °C, 20 min (**d**), 121 °C, 30 min (**e**) are in comparison with untreated control cells (**a**).

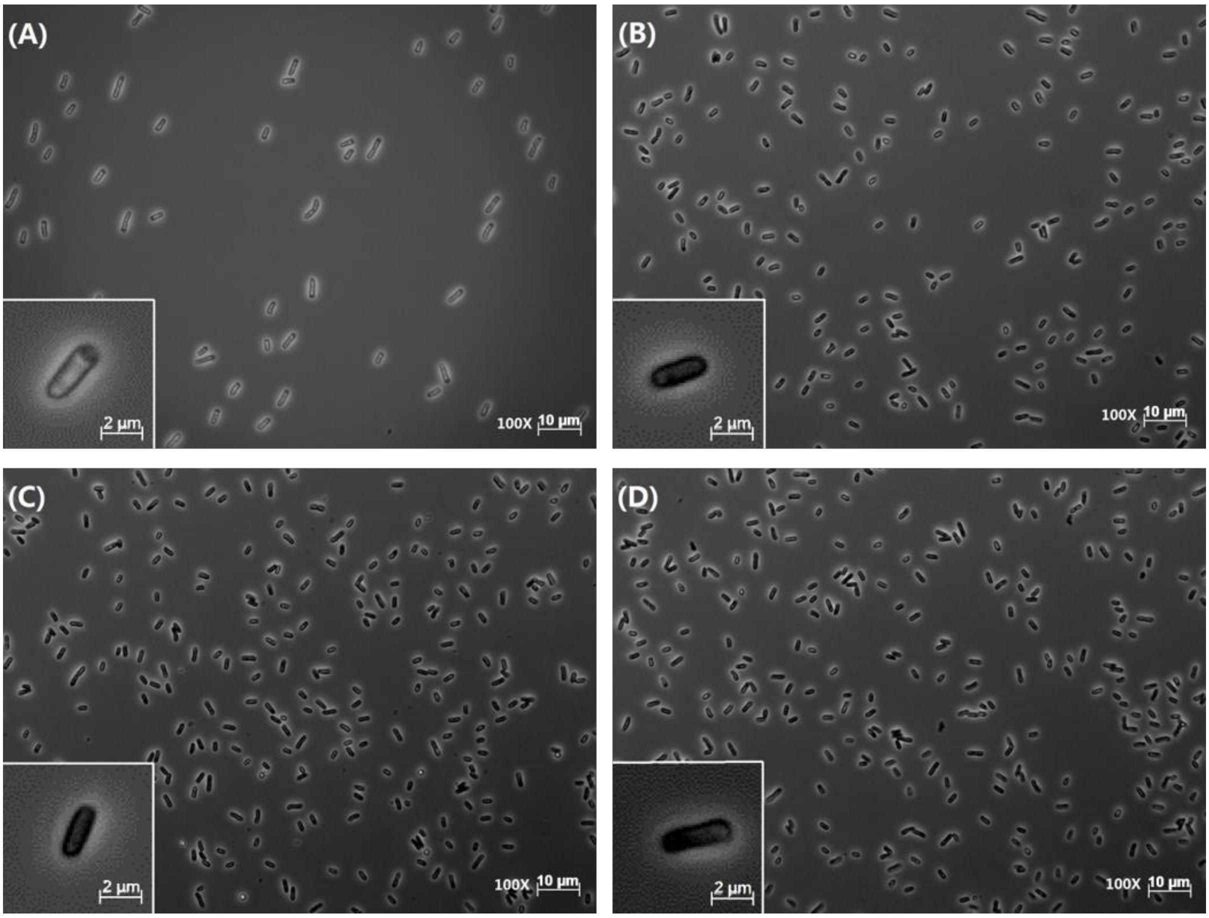

Figure S5. Microscopic pictures of *E. coli* cells treated with various concentrations of glutaraldehyde after 20 min incubation time. Cells affected by 100 mg L^{-1} (**b**), 200 mg L^{-1}(**c**), and 300 mg L^{-1} (**d**) of glutaraldehyde are in comparison with untreated control cells (**a**). The morphological structure of single cells treated with each dose of glutaraldehyde are shown at bottom left.

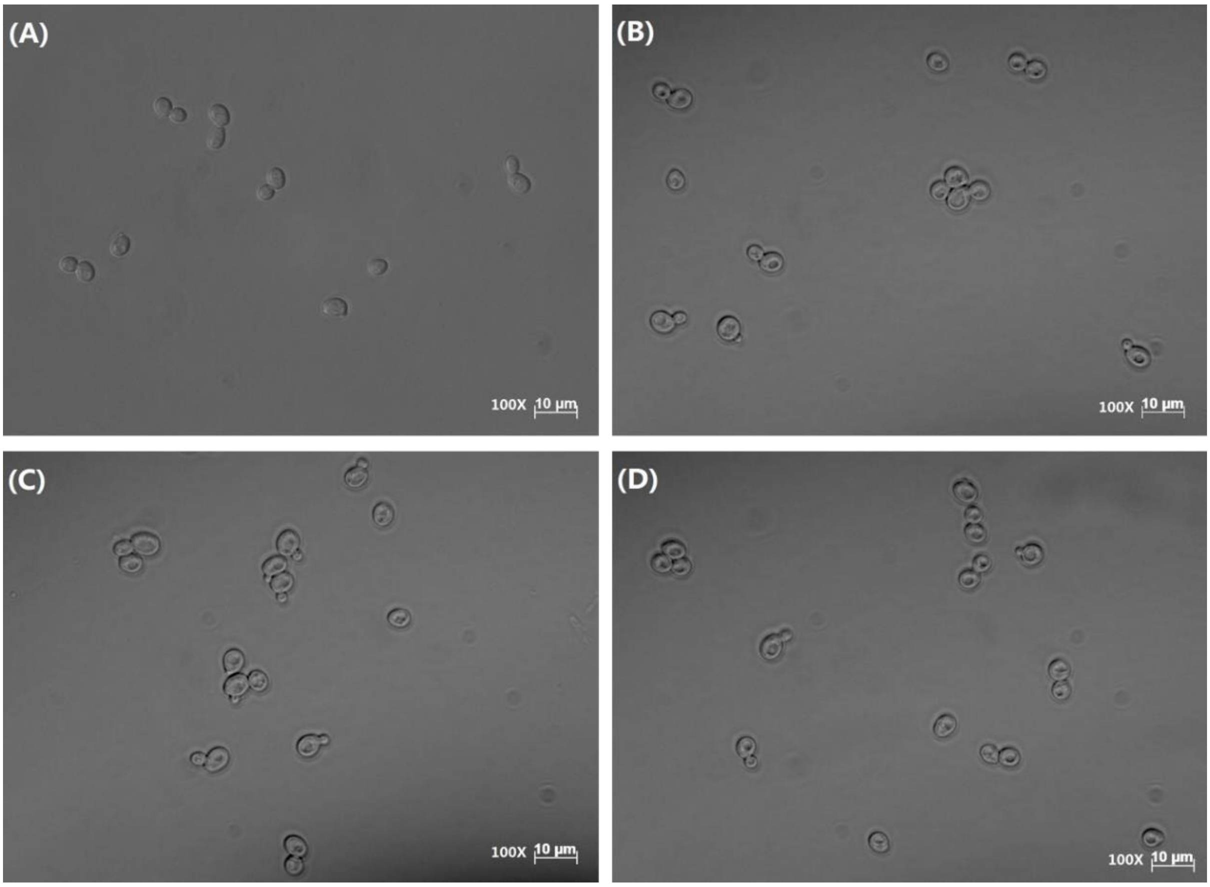

Figure S6. Microscopic pictures of *S. cerevisiae* cells treated with various concentrations of glutaraldehyde after 20 min incubation time. Cells affected by 100 mg L^{-1} (**b**), 200 mg L^{-1} (**c**), and 300 mg L^{-1} (**d**) of glutaraldehyde are in comparison with untreated control cells (**a**).

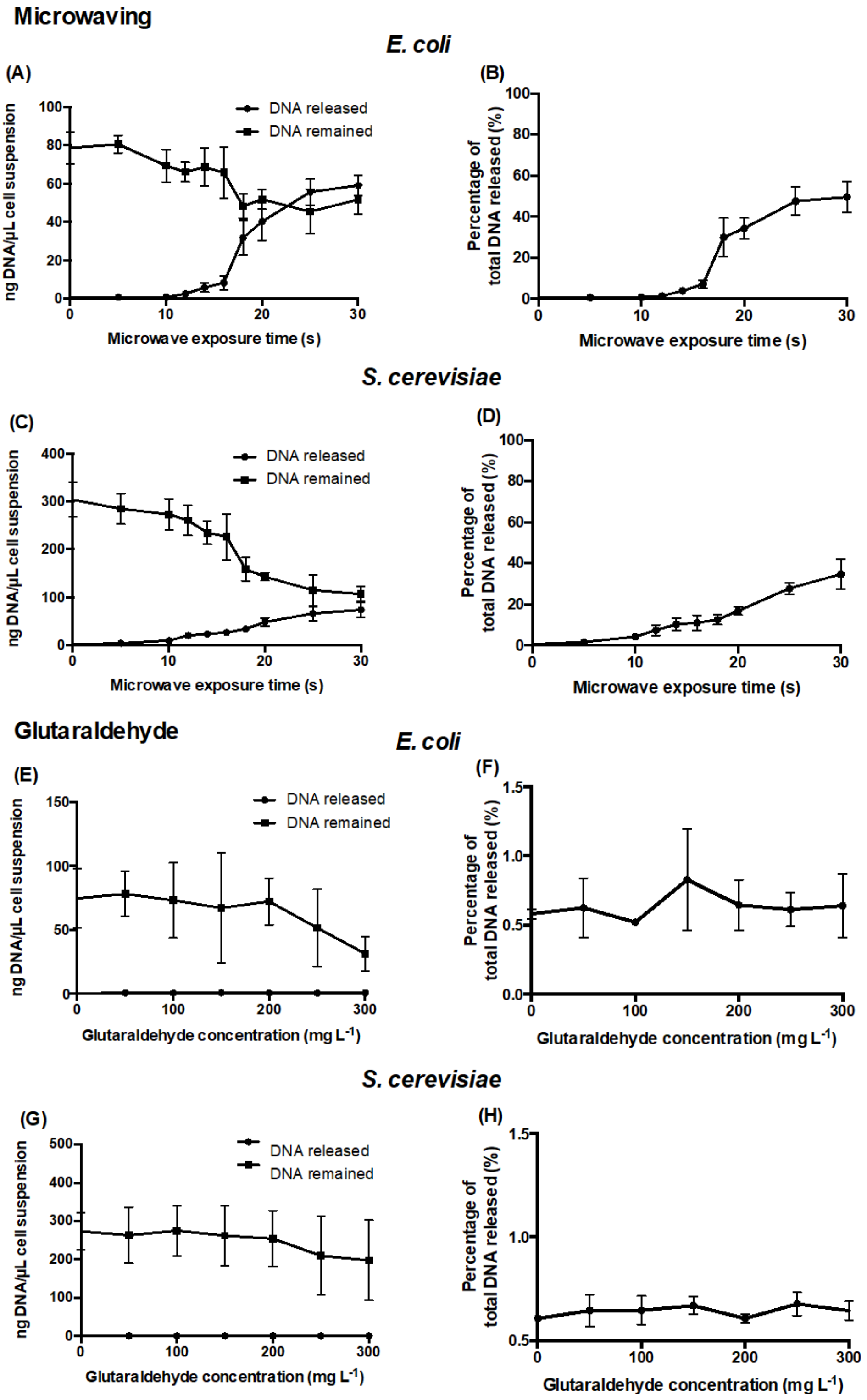

Figure S7. DNA quantification measurement on the amount of DNA released and remained of *E. coli* (A) and *S. cerevisiae* (C) treated with different microwave exposure times. DNA released and remained of *E. coli* (E) and S. cerevisiae (G) treated with different glutaraldehyde concentrations. Total DNA released from *E. coli* (B) and *S. cerevisiae* (D) treated with different microwave exposure times. Total DNA released from *E. coli* (F) and *S. cerevisiae* (H) treated with different glutaraldehyde concentrations. The percentage shows the ratios of the amount of DNA released in the supernatant against the total amount of DNA (released and remained combined).

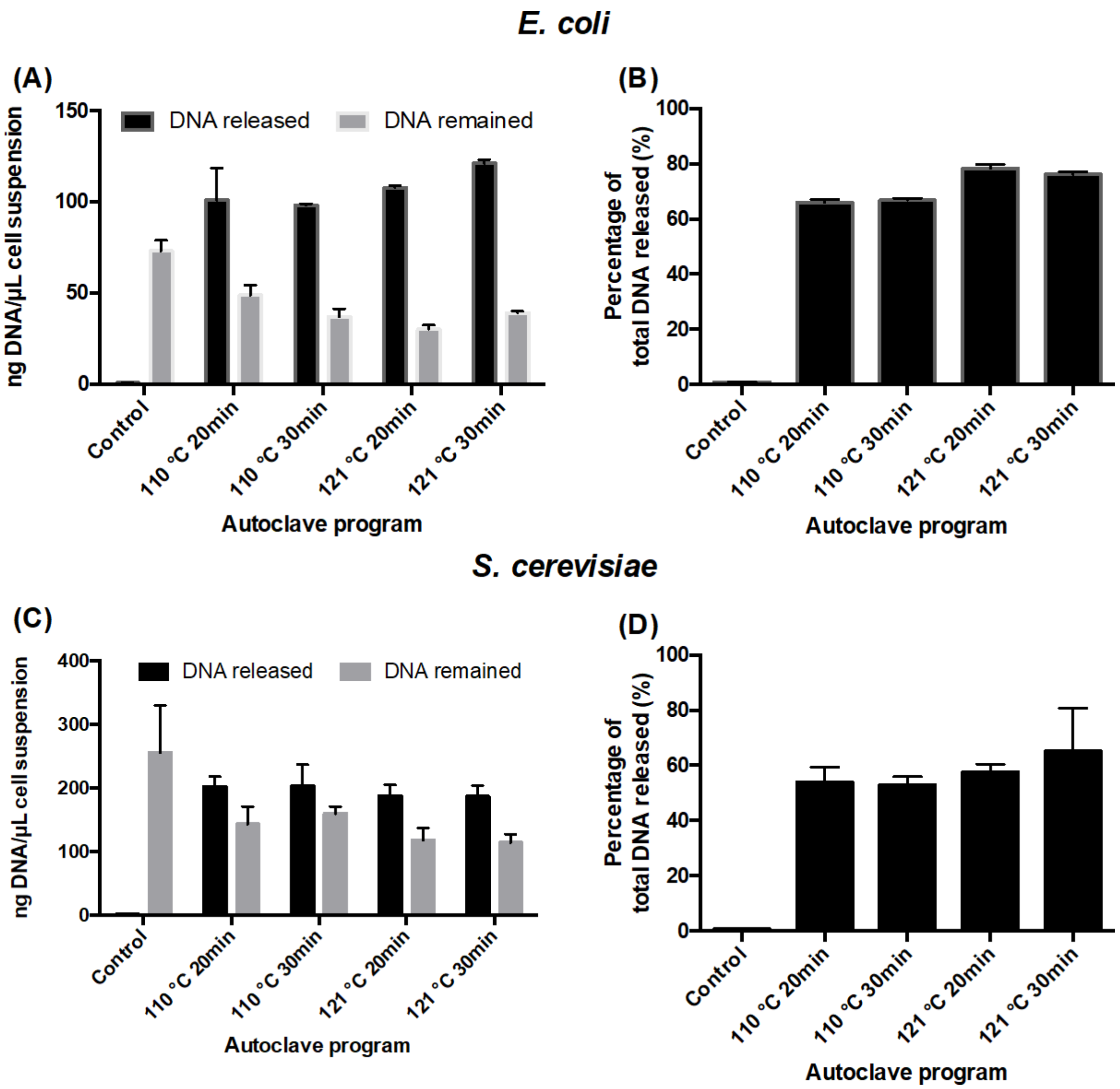

Figure S8. DNA quantification measurement on the amount of DNA released and remained of *E. coli* (A) and *S. cerevisiae* (C) treated with four different autoclaving programs. Total DNA released from *E. coli* (B) and *S. cerevisiae* (D) treated with four different autoclaving programs. The percentage shows the ratios of the amount of DNA released in the supernatant against the total amount of DNA.

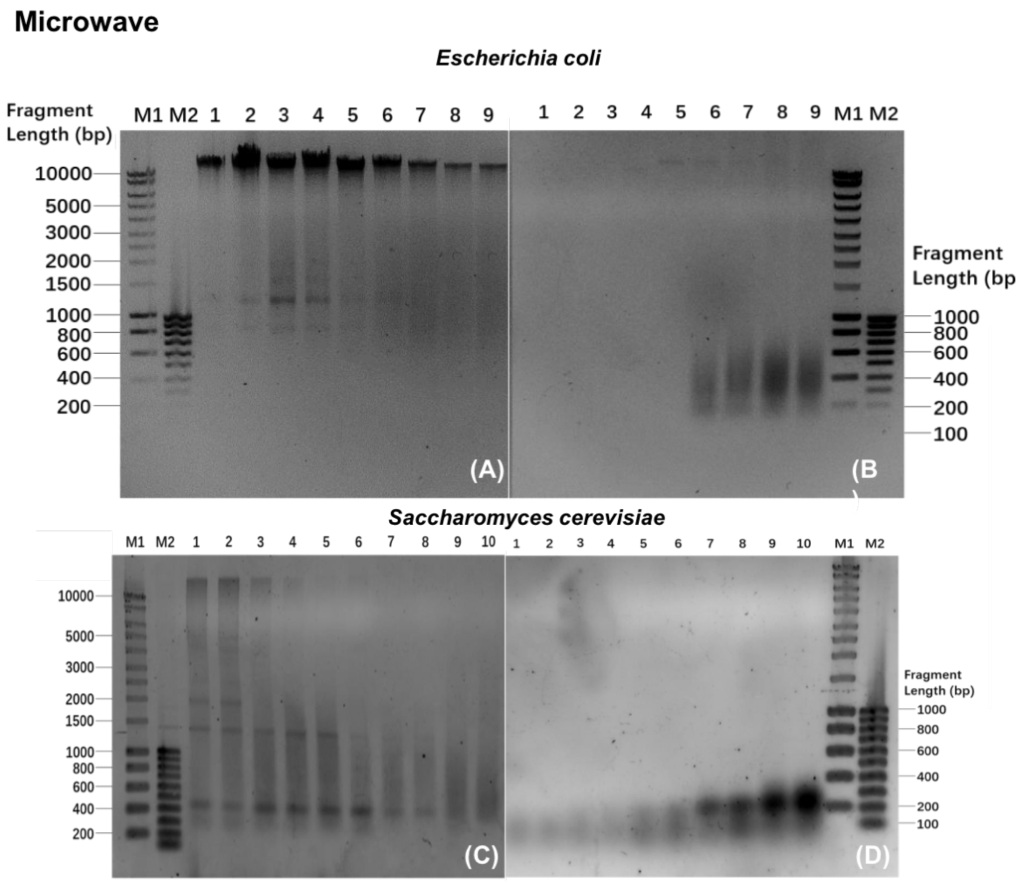

Figure S9. Electrophoretic gel of *E. coli* intracellular (A) and released DNA (B) together with *S. cerevisiae* intracellular (C) and released DNA (D) with increasing microwave exposure times. Lanes 1-10: *S. cerevisiae* intracellular DNA collected at 0, 10, 12, 14, 16, 18, 20, 25, 30, 40 s. Lanes 1-9 *E. coli* intracellular DNA collected at 0, 10, 12, 14, 16, 18, 20, 25, 30, 40 s.

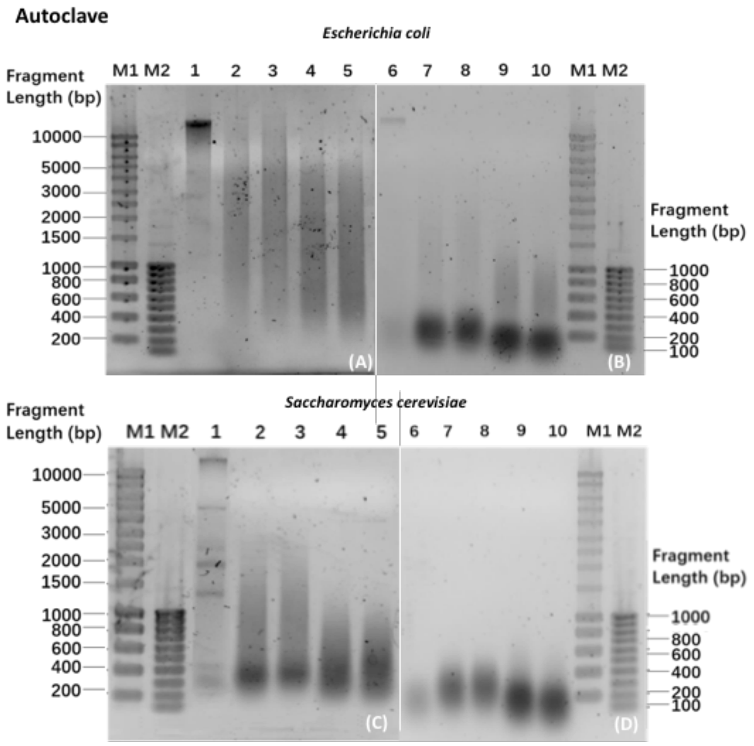

Figure S10. Electrophoretic gel of *E. coli* intracellular (A) and released DNA (B) together with *S. cerevisiae* intracellular (C) and released DNA (D) with different types of autoclaving. Lane 1: control sample intracellular DNA. Lane 2-5: intracellular DNA treated with P1, P2, P3 and P4. Lane 6: control sample released DNA. Lane 7-10: released DNA treated with P1, P2, P3 and P4 autoclaving programs. Autoclave programs: P1 (110 ºC – 20 min), P2 (110 ºC – 30 min), P3 (121 ºC – 20 min) and P4 (121 ºC – 30 min).

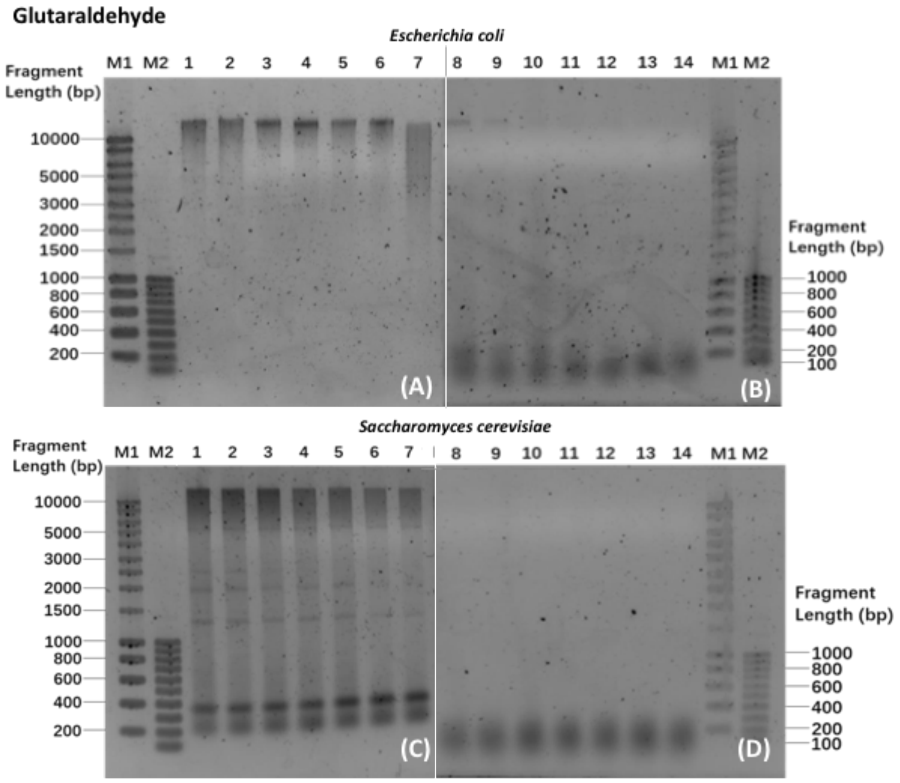

Figure S11. Electrophoretic gel of *E. coli* intracellular (A) and released DNA (B) together with *S. cerevisiae* intracellular (C) and released DNA (D) with increasing concentration of glutaraldehyde. Lanes 1-7: intracellular DNA collected at 0, 5, 100, 150, 150, 200, 300 mg L^{-1} glutaraldehyde. Lanes 8-14: released DNA collected at 0, 50, 100, 150, 200, 300 mg L^{-1} glutaraldehyde.
